# Supplementary material for: Genomic insights into the 2022–2023Vibrio cholerae outbreak in Malawi
Source: Nat Commun. 2024 Jul 26;15:6291. doi: 10.1038/s41467-024-50484-w (PMC11282309; doi:10.1038/s41467-024-50484-w)
Supplement: Supplementary file 3 — Description of Additional Supplementary Files [file 41467_2024_50484_MOESM3_ESM.pdf]

Description of Additional Supplementary Files

File Name: Supplementary Data 1
Description: Summary of all the *Vibrio cholerae* isolates from Malawi and globally used in this study (Excel file).

File Name: Supplementary Data 2
Description: Summary of the serogroups and presence and absence of genes in the 2022–2023 *Vibrio cholerae* isolates from Malawi (Excel file).
