# Supplementary material for: Genomic insights into the 2022–2023Vibrio cholerae outbreak in Malawi
Source: Nat Commun. 2024 Jul 26;15:6291. doi: 10.1038/s41467-024-50484-w (PMC11282309; doi:10.1038/s41467-024-50484-w)
Supplement: Supplementary file 6 — Reporting Summary [file 41467_2024_50484_MOESM6_ESM.pdf]

nature portfolio

Corresponding author(s): Chrispin Chaguza

Last updated by author(s): Apr 5, 2024

Reporting Summary

Nature Portfolio wishes to improve the reproducibility of the work that we publish. This form provides structure for consistency and transparency in reporting. For further information on Nature Portfolio policies, see our Editorial Policies and the Editorial Policy Checklist.

Statistics

For all statistical analyses, confirm that the following items are present in the figure legend, table legend, main text, or Methods section.

n/a	Confirmed	
☐	☒	The exact sample size (*n*) for each experimental group/condition, given as a discrete number and unit of measurement
☐	☒	A statement on whether measurements were taken from distinct samples or whether the same sample was measured repeatedly
☒	☐	The statistical test(s) used AND whether they are one- or two-sided<br>*Only common tests should be described solely by name; describe more complex techniques in the Methods section.*
☒	☐	A description of all covariates tested
☒	☐	A description of any assumptions or corrections, such as tests of normality and adjustment for multiple comparisons
☒	☐	A full description of the statistical parameters including central tendency (e.g. means) or other basic estimates (e.g. regression coefficient) AND variation (e.g. standard deviation) or associated estimates of uncertainty (e.g. confidence intervals)
☒	☐	For null hypothesis testing, the test statistic (e.g. *F*, *t*, *r*) with confidence intervals, effect sizes, degrees of freedom and *P* value noted<br>*Give P values as exact values whenever suitable.*
☒	☐	For Bayesian analysis, information on the choice of priors and Markov chain Monte Carlo settings
☒	☐	For hierarchical and complex designs, identification of the appropriate level for tests and full reporting of outcomes
☒	☐	Estimates of effect sizes (e.g. Cohen's *d*, Pearson's *r*), indicating how they were calculated

Our web collection on statistics for biologists contains articles on many of the points above.

Software and code

Policy information about availability of computer code

Data collection	No software was used for data collection. All the samples used in this study were collected by trained clinicians and processed by an experienced molecular microbiology team. The extracted DNA underwent whole genome sequencing at the Next Generation Sequencing Unit and Division of Virology at the University of the Free State, and the resulting data was analysed using the open-source tools listed below.
Data analysis	All software used in the analysis is freely and publicly available. We used cutadapt (version v4.4) [https://github.com/marcelm/cutadapt] to trim adapters from the raw sequence reads. Genome assembly was done using SPAdes (v3.13.1) [https://github.com/ablab/spades]. Whole-genome alignment was done using Snippy (version 4.6.0) using the "--ctgs" option [https://github.com/tseemann/snippy]. To generate the whole-genome alignment, we mapped the sequence reads of each Vibrio cholerae isolate against a merged reference sequence of O1 biovar El or strain N16961 chromosome 1 (GenBank accession: AE003852) and 2 (GenBank accession: AE003853) separated by ambiguous bases (Ns). We also included Vibrio mimicus genome Y4 strain (GenBank accessions: CP077425 and CP077426) in the alignment, which was used as an outgroup to root the phylogenetic tree. Before the whole-genome analysis, the location of known pathogenicity isolates and mobile genetic elements were identified using BLASTN (version 2.12.0+) [https://blast.ncbi.nlm.nih.gov/doc/blast-help/downloadblastdata.html]. We then masked the genomic regions containing pathogenicity islands, prophages, and ICEs using a custom Python script developed at the Wellcome Sanger Institute [https://github.com/sanger- pathogens/remove_blocks_from_aln]. We identified single nucleotide polymorphism (SNP) positions in the whole-genome alignment using snp-sites (version 2.5.1) [https://github.com/sanger-pathogens/snp-sites]. We excluded alignment positions based on the frequency of gaps using trimAl (version 1.4.rev15) [https://github.com/inab/trimal]. We constructed a maximum likelihood core-genome phylogeny of the Malawi Vc isolates using IQTREE (version 2.0.3) [https://github.com/Cibiv/IQ-TREE]. We used the "root" function in the APE package (version 5.6.2) to root the phylogenetic tree [https://cran.r-project.org/web/packages/ape/index.html]. We generated a ladderised tree using "ladderize" function in APE (version 5.6.2). Prunned trees of a subset of the isolates were generated by excluding other isolates from the phylogeny using the "drop.tip" function in APE (version 5.6.2). We visually explored the resulting phylogenetic tree using Taxonium (https://taxonium.org/) and Microreact (https://microreact.org/). Additional visualization of the

phylogeny in R was done using APE package (version 5.6.2). We counted the number of SNP differences between the Vibrio cholerae isolates using snp-dists (version 0.7.0) (https://github.com/tseemann/snp-dists). We detected the presence and absence of antimicrobial resistance genes in the Vibrio cholerae isolates using ABRicate (version 1.0.1) (https://github.com/tseemann/abricate). We compared each genome to a reference database of genes obtained from the NCBI AMRFinderPlus database and the virulence factor database (VFDB; http://www.mgc.ac.cn/VFs/). We used a custom database of the genes used for biotyping, AMR, and pandemic lineage and species identification in the CholeraeFinder tool (https://cge.cbs.dtu.dk/services/CholeraeFinder). We also inferred the genotypic AMR of the Vc isolates using VibrioWatch implemented in the PathogenWatch web tool (https://pathogen.watch/). We mapped nucleotide sequence k-mers of each isolate against all known reference lipopolysaccharide (LPS) O-antigen biosynthesis gene cluster sequences (PubMed ID: 35930328), using KMA (version 1.4.12a) [https://anaconda.org/bioconda/kma], to determine the specific serogroups and serotypes of the Vibrio cholerae isolates. The following options were used when running KMA: "-ef -dense -ex_mode -mct 1.0 -1t1 -mrs 0.1". We also used KMA to identify the presence and absence of the mobile genetic elements. Comparison of the mobile genetic sequences was done using BLASTN (version 2.12.0+) and ACT (version 18.1.0) [https://anaconda.org/bioconda/artemis]. The wbeT sequences were extracted from the Inaba and Ogawa Vc isolates using BLASTN (version 2.12.0+) [https://ftp.ncbi.nlm.nih.gov/blast/executables/blast+/LATEST/] and "getfasta" option implemented in bedtools (version 2.30.0) [https://github.com/arq5x/bedtools2]. Recombinogenic regions and maximum likelihood phylogeny of the 7PET strains was generated using Gubbins (version 3.2.1) [https://github.com/nickjcroucher/gubbins]. Phylogenetic trees were generated using ggtree (version 3.10.0) [https://guangchuangyu.github.io/software/ggtree/].

For manuscripts utilizing custom algorithms or software that are central to the research but not yet described in published literature, software must be made available to editors and reviewers. We strongly encourage code deposition in a community repository (e.g. GitHub). See the Nature Portfolio guidelines for submitting code & software for further information.

Data

Policy information about availability of data

All manuscripts must include a data availability statement. This statement should provide the following information, where applicable:

- Accession codes, unique identifiers, or web links for publicly available datasets
- A description of any restrictions on data availability
- For clinical datasets or third party data, please ensure that the statement adheres to our policy

The whole-genome sequencing data for the study isolates has been deposited in the European Nucleotide Archive (ENA). The accession numbers and other isolate metadata are provided in the Supplementary Data 1 file. All the other data supporting the findings of this study are described in this paper or are available as part of the supplementary material.

Research involving human participants, their data, or biological material

Policy information about studies with human participants or human data. See also policy information about sex, gender (identity/presentation), and sexual orientation and race, ethnicity and racism.

Reporting on sex and gender	This study used Vibrio cholerae isolates sampled from patients of any sex who had cholera. The isolates were phenotyped to confirm the species. Sex and gender of the patient was not used in the analysis, as this information was not relevant to address the study objectives.
Reporting on race, ethnicity, or other socially relevant groupings	This study used Vibrio cholerae isolates sampled from patients of any race or ethnicity in Malawi (almost all black Africans/ Malawians) who had cholera. We did not collect information on the specific social groupings of the patients. The isolates were phenotyped to confirm the species. Data on race, ethinity or other socially relevant groupings of the patients was not used in the analysis, as this information was not relevant to address the study objectives.
Population characteristics	With the exception of one environmental isolate, all the Vibrio cholerae isolates were collected from patients who had cholera in Malawi. We only used remnant stool samples collected from the cholera patients. Detailed information about the age, past treatments, and genotypic information was not available for this study.
Recruitment	The patients whose stool samples were included in this study were selected based on having cholera-like symptoms.
Ethics oversight	This work was conducted according to the guidelines of the Declaration of Helsinki and was approved by the National Health Sciences Research Committee, Lilongwe, Malawi (Protocol #867) and the Research Ethics Committee of the University of Liverpool, Liverpool, UK (000490) under the Diarrhoea Surveillance study, and the College of Medicine Ethics Committee (COMREC, Protocol #P.10/22/3790) under the NIHR Global Health Research Group on Gastrointestinal Infections: Facilitating the Introduction and Evaluation of Vaccines for Enteric Diseases in Children in Eastern and Southern sub-Saharan Africa study.

Note that full information on the approval of the study protocol must also be provided in the manuscript.

Field-specific reporting

Please select the one below that is the best fit for your research. If you are not sure, read the appropriate sections before making your selection.

☒ Life sciences ☐ Behavioural & social sciences ☐ Ecological, evolutionary & environmental sciences

For a reference copy of the document with all sections, see nature.com/documents/nr-reporting-summary-flat.pdf

Life sciences study design

All studies must disclose on these points even when the disclosure is negative.

Sample size	Since surveillance of Vibrio cholerae, especially the preservation of clinical isolates is not routinely undertaken in all districts in Malawi, we could not systematically select representative samples for microbiological examination. Therefore, we used a convenience sampling method.
Data exclusions	We sequenced 75 isolates. Of these, 68 were successful. After further quality control to exclude potentially contaminated samples, we remained with 45 genotypically confirmed Vibrio cholerae isolates for this analysis.
Replication	Replication of the study using an independent dataset was not done.
Randomization	This study focused on patients with suspected cholera diarrhoea. No randomisation into treatment groups was done. Our analysis is primarily descriptive, therefore, we did not do statistical analyses to compare any treatment groups.
Blinding	No blinding was performed in this study. Blinding was considered not to be necessary for our surveillance study as the nature of the study was observational and no intervention was given to the patients. Therefore, there was no risk for bias by not blinding the investigators.

Reporting for specific materials, systems and methods

We require information from authors about some types of materials, experimental systems and methods used in many studies. Here, indicate whether each material, system or method listed is relevant to your study. If you are not sure if a list item applies to your research, read the appropriate section before selecting a response.

Materials & experimental systems

n/a	Involved in the study	
☒	☐	Antibodies
☒	☐	Eukaryotic cell lines
☒	☐	Palaeontology and archaeology
☒	☐	Animals and other organisms
☒	☐	Clinical data
☒	☐	Dual use research of concern
☒	☐	Plants

Methods

n/a	Involved in the study	
☒	☐	ChIP-seq
☒	☐	Flow cytometry
☒	☐	MRI-based neuroimaging
